# The High School to University Transition: Exploring the interplay of territory, socioeconomic factors, and gender dynamics

**DOI:** 10.1371/journal.pone.0326510

**Published:** 2025-10-24

**Authors:** Andrea Priulla, Martina Vittorietti, Vincenzo Giuseppe Genova, Massimo Attanasio

**Affiliations:** 1 Department of Economics and Law, University of Enna KORE, Enna, Italy; 2 Department of Applied Mathematics, Delft University of Technology, Delft, The Netherlands; 3 Department of Economics, Business, and Statistics, University of Palermo, Palermo, Italy; Public Library of Science, UNITED KINGDOM OF GREAT BRITAIN AND NORTHERN IRELAND

## Abstract

Education inequalities persist globally, particularly in Italy, often influenced by factors beyond student ability. This paper aims to study the pure territory effect on education inequalities controlling for other factors such as gender, socioeconomic status, and high school performance. As in a clinical study, we evaluate the effect of multiple treatments represented by territorial clusters on the students’ enrolment choices. The inverse of propensity score estimates obtained with a gradient-boosted model (GBM) procedure are used as weights of a multinomial logistic regression model to evaluate the probability of enrolling at the university inside or outside their own place of residence. The study highlights the importance of considering the complex interplay between territory and economic variables in explaining inequalities in the transition from high school to university. Especially in northern regions, we show that the territorial effect on mobility choices, was covered by socioeconomic status and previous educational attainment. In the southern regions, the results highlight a more pronounced territorial heterogeneity regarding the choice to move to another region compared to northern regions.

## 1 Introduction

Equity in student achievement aligns with the principle of equality of opportunity, which means that academic success should not be influenced by factors outside the individual’s control [1]. Ideally, any disparities in academic performance between students should solely stem from differences in their abilities or hard work. Nevertheless, there are significant inequalities in academic achievement that are correlated with students’ background characteristics, such as gender and socioeconomic status, high school careers, and the territorial context. The Organisation for Economic Co-operation and Development (OECD) has established a consistent method for assessing educational equity in its countries [2] and has published a report on the factors influencing the quality and equity of education. The PISA results highlighted persistent and substantial educational inequalities, with advantaged students consistently outperforming their disadvantaged peers across all OECD countries.

In Italy, several studies have explored the great influence of the territory on educational achievement at a macro-level [3,4]. Geographical location can unfairly contribute to disparities in access to higher education on a large scale [5].

The South-North dualism represents an unending inequity affecting the Italian territory, mirrored in the higher education system in a vicious circle affecting social and economic development and boosting the brain drain from southern regions, that are already plagued by significant school dropout rates and low tertiary education enrollment.

Yet, concentrating exclusively on territory may ignore the impact of the socioeconomic aspects such as gender and parental background.

It is challenging to isolate the impact of single unequal factors on educational outcomes, as these factors are often interrelated and can interact in complex ways. In this framework, intersectionality should be a conceptual aspiration and a research imperative for education researchers [6].

The aim of this paper is to study the effect of the so-called “territorial identity” [7] of the students on enrolment pathways. In detail, we want to single out the territory’s contribution in the transition from high school to university, controlling for student sociodemographic characteristics and previous high school performance. Students have three options during this transition: not enrolling at a university, enrolling locally, or enrolling in another region.

At the individual level, territorial identity often acts as both a push and pull factor. On the one hand, a strong attachment to the local community and cultural heritage reinforces the desire to remain, despite economic pressures or limited opportunities. In this context, the role of family ties and obligations contribute to the decision to stay, especially in Southern Italy. This attachment can also influence educational pathways, as some individuals may prioritize remaining close to home over pursuing higher education, opting instead to integrate into local employment opportunities. This choice can be perceived as a way to maintain a connection to the community, even at the cost of personal advancement.

On the other hand, the same strong territorial identity can amplify the frustration of perceived stagnation, driving migration as a way to escape socioeconomic constraints and seek personal fulfilment [8]. Southern youth, in particular, often feel torn between loyalty to their hometowns and the ambition to achieve professional success, which is strongly associated with the choice to enroll in a Northern university [9].

As far as the author knowledge, this is the first attempt to study the territorial effect at a micro-level due to the lack of administrative data.

In this paper, we use micro-data from two Italian administrative sources: Anagrafe Nazionale Studenti (ANS) and the National Evaluation Institute for the School System (INVALSI). In particular, we consider the cohort of students enrolled on grade 13 (last year of high school in Italy) in the academic year 2018/19, for which we have complete data on the first year at university in 2019/20.

To isolate the territory effect, we carry out a propensity score analysis in which we aim to balance the gender, SES, maths test scores and the high school curriculum distribution of clusters of 4 regions’ municipalities: Veneto, Tuscany, Apulia, and Sicily. We conduct a balanced analysis at the regional level and at the same time obtain results valid for the whole country. In this regard, the use of propensity scores to control for pretreatment imbalances on observed variables in non-randomized or observational studies examining the causal effects of treatments or interventions has become widespread over the past decade [10]. Most studies that use propensity scores to control for imbalance compare just two treatment groups of interest (e.g., treatment and control). Nonetheless, several papers have shown that propensity score methods can be extended to the multiple-treatment case [11]. In this paper, we use a multi-treatment propensity score approach based on gradient-boosted models (GBM) as proposed in [12]. The inverse of the propensity score estimates are then used as weights of a multinomial logistic regression model that describes i) the probability of being stayer *vs* the probability of not enrolling at university and ii) the probability of enrolling in a university outside the region where the student attended high school *vs* the probability of not enrolling at university.

This paper aims to answer two main research questions: what is the effect of territory on the enrolment choices of Italian graduates? Is this effect confounded by other factors such as gender, socioeconomic status and high school performance?

The outline of the paper is as follows: in Sect 2, the educational inequalities theoretical framework is presented; in Sect 3, the data sources and the main variables used in the analysis are presented; in Sect 4, we propose and describe a new statistical approach, starting with the construction of clusters of municipalities, we then introduce the balancing procedure based on GBM and finally we present the final model used to evaluate the outcome of interest; in Sects 5 and 6, we present both preliminary and final results; finally, discussion, conclusions and future developments can be found in Sect 7.

## 2 Theoretical framework

Educational inequalities in academic achievement, though should be attributed exclusive to the competences of the students are often correlated and sometimes uniquely determined by students’ background characteristics, such as gender and socioeconomic status, high school careers, and the territorial context. The impact of family socioeconomic status (SES) on children’s academic achievement has been extensively studied [13]. The intensity of the relationship between family SES and educational achievements has been shown to vary across countries due to differences in education systems and societal changes [14]. According to [15] and [16], students’ social origin is the main factor contributing to educational disparities. Based on [17] insights, families play a role in shaping their children’s “habitus" – a set of perceptions and expectations about life opportunities, including education. [18] have empirically demonstrated how habitus significantly influences the college choices of Hispanic students in the United States, affecting both enrollment decisions and the selection of specific institutions. When families and local communities encourage higher education, students often gravitate towards institutions where Hispanic peers are already enrolled. [19] provide additional evidence from the UK that highlights the alienation experienced by working-class students at prestigious universities. This feeling arises from their struggle to adapt to an unfamiliar academic environment while reconciling it with their backgrounds. As a result, they undergo a shift in their habitus. This dynamic highlights habitus as a mechanism that can perpetuate social inequalities, a point further reinforced by [20], who identifies a strong link between social origins and internal educational migration in Italy. Building on Bourdieu’s theoretical framework, [21] introduces the concept of “migration-facilitating capital", which encompasses financial and social resources alongside habitus. Similarly, [22] uses a Bourdieusian lens to explore how school choice can lead to spatial segregation, with higher-income families gravitating toward so-called “prestigious" schools, while working-class families remain concentrated in other institutions. Habitus also shapes aspirations, as illustrated by [23] aspiration-capability framework, which provides insights into decisions regarding both mobility and immobility. The findings of [24] reveal that for Neapolitan students, pursuing a university degree is not perceived as a direct pathway to better job prospects. When individuals envisioned a future where employment matched their degree, their willingness to relocate increased, revealing an attachment to their place of origin. Furthermore, the authors argue that examining mobility in disadvantaged contexts reveals a complex interplay between present circumstances and future aspirations. Thus, the connection between family background and schooling choices is particularly strong [16,25]. In the work of [26], authors show that capable students from underprivileged families face limitations in their educational choices, especially for university enrollment, where factors such as expenses and quality have a greater impact on students from lower socioeconomic backgrounds.

Also gender contributes to educational inequalities [27–29]. In the early part of the 20^*th*^ century, gender played a significant role in determining educational achievement. This was primarily due to institutional factors that restricted women’s access to post-secondary education in the United States, cultural factors that discouraged women from pursuing higher education, and social factors that emphasized women’s roles as primary caregivers [30]. Female educational attainment has increased substantially since the II World War and now exceeds that of males in secondary and tertiary education in many European countries [31,32]. In Italy, females have overcome males in high school attendance since 1981, and the same has been recorded in university attendance since 1989. Nevertheless, the gender composition at both high school and university levels in the different educational fields is highly heterogeneous. In 2018, there was a higher representation of females in the humanities track (*liceo classico*, 69% of females), which shares a similar university orientation with the scientific track (*liceo scientifico*, 45%) but has fewer hours devoted to scientific subjects [33]. Females are also largely over-represented in another humanistic track (*liceo delle scienze umane*, 84%) and underrepresented in technical and vocational schools, particularly those with an industrial focus (19%), which prioritize market-driven career prospects. At the university level, male students are more likely to enrol in Science, Technology, Engineering, and Mathematics (STEM) degrees, while females are more interested in humanistic and non-STEM fields in general [34].

The influence of both socioeconomic status and gender on education inequalities is enhanced by the structure of the Italian high school system. In fact, a widely held belief is that a highly tracked high school system undermines equality of opportunity by strengthening the role of parental privilege [35]. In Italy, the school and university system has had a uniform and centralized structure since the unification of Italy and was further reinforced during the fascist era. Disparities in the system have been significant since unification. For example, in 1968, the illiteracy rates in the South were still notably higher, with a 23% repetition rate in first grade in the primary school in Calabria compared to 9% in Veneto [36]. Another concerning data point pertains to the early school dropout rates (in Italy, the school leaving age is 16), which, on average, were slightly below 10% in 2021, with pronounced disparities between the Northern and Southern regions. In fact, the rates are approximately 20% in some Southern regions, such as Campania, Sardinia, and Sicily, while they remain below 5% in most northern regions [37]. In the last 30 years, educational institutions have experienced a slight degree of autonomy in their teaching offerings and organizational structure, and they have had the opportunity to access external funding at the regional level and compete for national public funding in various forms.

Several studies have explored the great influence of the territory on educational achievement at a macro-level [3,4]. These studies have revealed that historical inequalities in economic growth and development among different regions have contributed to disparities in the effectiveness and efficiency of the education system. They have also emphasized the significance of the region effect in the north-south axis, which is a major determinant of the unequal distribution of educational opportunities across the nation [38–40]. Furthermore, INVALSI indicates that students residing in Southern Italy tend to achieve lower scores on standardized assessments in subjects such as Italian, Mathematics, and English compared to their peers in the North, regardless of the educational level [41]. The persistent social inequalities characterizing the Southern regions of Italy encompass both quantitative differences (e.g., illiteracy rates and years of education) and qualitative distinctions (e.g., in terms of differential access to higher status courses), reflecting their relative educational disadvantages [42,43]. Hillman’s theory of educational deserts [44] suggests that limited access to higher education is both a geographic and economic issue. In areas with weak labor markets and high unemployment, the perceived value of a university degree decreases, creating a significant economic barrier to enrollment, thereby exacerbating socio-economic divides and limiting upward mobility. The work of [26] has highlighted that university enrolments in Italy are certainly lower in areas with high youth unemployment rates, such as the southern Italian regions, showing that when labour market perspectives are poor, discouragement to attend higher education is common.

In the past decade, encouraging signs have emerged alongside critical elements. There has been an improvement in educational attendance and the overall efficiency of the Italian university system, with a reduction in disparities among universities in different regions [45]. Nevertheless, relevant critical disparities remain that appear to be determined mainly by contextual factors over which universities have limited control. The basic school system, beneath the apparent uniformity of a national model, is increasingly marked by profound disparities in the provision and distribution of schools and teachers across the territory. These differences are certainly linked to the characteristics of the area and the structure of the population, but they also result from policies inspired by the rationalization of resource utilization that have affected the quality of life of individuals, often reducing and, not infrequently, zeroing out the provision of educational and healthcare services. This heterogeneity in educational offerings and, more generally, other citizen services, overlaps with geographical inequalities determined by the economic structure, road networks, and transportation, the availability of other cultural institutions (libraries, museums, theatres, etc.), and, not least, the socio-demographic characteristics of the inhabitants [46]. The major manifestation of the territorial inequality is the decrease in university enrolments in the South. This occurred for two main reasons: the demographic shift, which is affecting the entire Italian territory, and the students’ mobility, which is unidirectional from South to Center-North [4,47,48].

## 3 Data

The empirical analysis reported in this work relies upon a linkage of micro-data coming from two Italian administrative archives:

- **INV-S**: micro-data from the *Istituto nazionale per la valutazione del sistema educativo di istruzione e di formazione* (INVALSI). INVALSI carries out national large-scale standardized tests to evaluate the overall quality of the educational system for each type of high school track. These tests are administered annually to students at five levels of education (grades 2, 5, 8, 10, and 13), aiming to evaluate mathematical and Italian language skills and, from 2018, English reading and listening skills. In addition, INVALSI collects information regarding students’ profiles such as sociodemographic status, socioeconomic status, geographical provenience, and further indicators of past school performance (i.e., whether the student had a regular high school career). Hence, it is possible to associate several important individual characteristics with academic performance.
- **ANS-U**: micro-level longitudinal data from the National Archive of University Students (ANS) [49]. This database includes the entire information about the university careers of all the students enrolled in Italian universities from 2008 to 2020. Every first-year student is recorded with information about his/her high school background and his/her entire university career.

The linkage of these databases allows a detailed investigation of the transition from high school to university at the individual and school level.

We consider the cohort of students attending grade 13 – corresponding to the last year of upper secondary school – in 2018/19 in Italy. Among them, we consider the two flows in 2019/20: not enrolled at university and enrolled at university. Unfortunately, ANS does not collect any information about students enrolling at universities abroad. For this reason, those students will be considered not enrolled. The covariates considered throughout the analysis are:

– The socioeconomic status (SES), expressed by the ESCS index (Index of Economic, Social and Cultural Status). It is a composite indicator used in the OECD PISA survey and INVALSI tests that measures the socioeconomic and cultural status of the students’ families. The PISA has traditionally built the ESCS index as a weighted average of three indices: parental educational attainment (in years), parental occupational status on the “International Socio-Economic Index” (ISEI) scale [50], and a measure of “household possessions” [51]. A negative (positive) value of the index indicates a lower (higher) SES than the Italian average;
  – The scores in INVALSI mathematics tests. Although INVALSI tests students’ abilities also in Italian and English reading and listening, we decided to focus on mathematics since it has been shown to be a strong predictor of university enrolment and success [25,33,52];
  – The type of high school curriculum, categorized in humanistic/scientific *licei*, other *licei*, and technical/vocational schools. In Italy, those curricula largely differ in their academic orientations, with humanistic and scientific *licei* considered the main routes for a university enrolment, followed by the other *licei*. Conversely, technical and vocational schools are considered the primary routes to the labour market;
  – Gender, to account for the differences in academic orientation between males and females.

## 4 Methods

In this section, an overview of the methods used for the analysis is presented. The approach can be summarized in three steps: *i*) cluster construction, *ii*) balancing procedure and *iii*) weighted regression model.

### 4.1 Cluster construction

The construction of clusters, as detailed in [53], is based on the idea that students within the same geographic area can communicate and potentially trigger a mobility process towards universities outside of their place of residence. The area of origin consists of several municipalities that are centred around a central hub municipality containing at least one high school. The cluster construction works as a compromise between the size of municipalities and provinces: municipalities are small and too many to capture mobility phenomena, while provinces are too large and heterogeneous.

The procedure can be summarized as follows:

1. construct an Origin-Destination matrix *OD*(*i*,*j*), where *i* represents the municipality of residence of the students and *j* is the municipality of the high school attended by the students;
2. within the list of *J* destinations, select the municipalities where schools have at least *n* students who will be future university freshmen. These municipalities are the hubs chosen as starting points for the determination of the areas of origin. To achieve a reasonable balance between the number of provinces and municipalities, a threshold was set to 200 students;
3. if *i* is a hub municipality, simply assign students from municipality *i* to hub *j* with *i* = *j*;
4. if municipality *i* is not a hub, identify j^*^, with $i\neq j^{*}$, as the municipality with the highest number of high school students living in *i*:
  - if j^*^ is hub ⇒ attribute the *i*–*th* municipality to j^*^;
  - if j^*^ is not a hub ⇒ assign students of the *i*-th municipality to the *j*-th hub municipality that is closest in terms of physical distance.
5. The aforementioned steps assigned students living in *k* non-hub municipalities $\left(C_{1},C_{2},\ldots ,C_{K}\right)$ to hub municipalities. The area of origin (AO) of a hub *j* is thereby defined as:
  $$AO(hub_{j})=hub_{j}\cup \left(\overset{K}{\underset{k=1}{⋃}}C_{k}\right)$$ (1)

Following this idea, the university choices made by students are primarily influenced by their area of origin and the relationships that they have with their high school and local communities, which could potentially trigger a mechanism of mobility.

### 4.2 Balancing procedure

The balancing procedure is based on propensity score analysis for multiple treatments. In recent years, the use of GBM has become increasingly popular in the field of causal inference. One common application of GBM is for the estimation of propensity score weights, which are used to balance covariate distributions between treatment groups in observational studies [12]. The accuracy and precision of propensity score estimation can be enhanced using GBM, which can handle complex and nonlinear connections between covariates. GBM is a modelling technique that fits a piecewise constant model to predict a binary outcome, such as a treatment indicator. The model consists of multiple simple regression trees that are iteratively combined to create an overall piecewise constant function. Adding too many trees can overfit the data, so an intermediate iteration is used to minimize external criteria, such as out-of-sample prediction error or covariate imbalance in the treatment and control groups for propensity score estimation. The algorithm starts with a single tree and adds new trees at each iteration to improve the fit to the residuals from the previous iteration. A scalar of less than one is then used to shrink the predictions from each tree to improve the smoothness of the model [54].

Here, we use GBM to balance the clusters within each of the four selected Italian regions according to a set of covariates. The procedure is here outlined.

Let *M*_*r*_ denote the number of treatments considered, in our case representing the clusters for the *r*-th region. Let **X** denote the matrix of *K* observed pretreatment covariates (in our case, gender, maths test scores, SES, and high school track). We create dummy indicators, $T_{zr}(t_{r})$ that is, $T_{zr}=t_{r}$ if individual *z* in region *r* was observed under treatment *t*_*r*_, where r=1,…,R, and $z=1,\ldots ,n_{r}$, with *R* the number of regions considered and *n*_*r*_ the number of individuals in each region. We fit separate GBMs to each dummy treatment indicator and obtain the estimated propensity score for the given treatment. Fitting GBM one treatment at a time produces propensity scores and corresponding inverse probability of treatment weights for individuals assigned to that particular treatment group. For each treatment indicator, the estimated propensity score, ${\hat{p}}_{z,t_{r}}({𝐗}_{z,t_{r}})$, computed from the iteration of the GBM fit, yields the ‘best balance’ between units with $T_{zr}(t_{r})$ and the pooled sample from all treatments. The optimal iteration is typically determined by stopping rules that attempt to choose the number of iterations that maximize the predictive performance of the model. Based on the model proposed by [12], we decided to use the stopping rule based on the absolute standardized mean difference (ASMD). ASMD equals the absolute value of the difference between the weighted mean for the treatment group and the weighted mean for the control group divided by the unweighted standard deviation of the pooled sample. The stopping rule based on ASMD involves selecting the iteration at which the maximum or mean ASMD across all covariates falls below a pre-specified threshold.

For each covariate *k*, ASMD can be formally expressed as


$$ASMD_{k,t_{r},t_{l}}=\frac{\left|{\bar{X}}_{k,t_{r}}-{\bar{X}}_{k,t_{l}}\right|}{{\hat{\sigma }}_{k,t_{r}(pooled)}},\;\;r,l=1,\ldots ,R,\;r\neq l$$

where


$${\bar{X}}_{k,t_{r}}=(\sum_{z=1}^{n_{r}}X_{k,t_{r}}/{\hat{p}}_{z,t_{r}}\left({𝐗}_{z,t_{r}})\right)/(\sum_{z=1}^{n_{r}}1/{\hat{p}}_{z,t_{r}}\left({𝐗}_{z,t_{r}})\right)$$

is the propensity score weighted mean of the covariate, ${\hat{p}}_{z,t_{r}}({𝐗}_{z,t_{r}})$ is the estimated propensity score for the treatment obtained from our GBM fits, and ${\hat{\sigma }}_{k,t_{r}(pooled)}$ denote the unweighted standard deviation of the covariate for the pooled sample across all treatments in a specific region *r* [12].

### 4.3 Weighted multinomial logistic regression models

In the last step, the inverse of the propensity score estimates are used as weights in a multinomial logistic regression model

In detail, we consider the following ratios: the first one is

$$log\left[\frac{\text{P(Y = Stayer)}}{\text{P(Y = Not Enrolled)}}\right]=\beta_{0_{r},1}+\beta_{t_{r},1}*t_{r}$$ (2)

that is the odd of enrolling at a university located in the same region of residence with respect to the one that did not enroll; the second one is

$$log\left[\frac{\text{P(Y = Mover)}}{\text{P(Y = Not Enrolled)}}\right]=\beta_{0_{r},2}+\beta_{t_{r},2}*t_{r}$$ (3)

that is the odd of enrolling at a university located in another region with respect to the one that did not enroll.

## 5 Preliminary analysis

In this section, we conduct a preliminary analysis to examine the differences between and within four Italian regions: Veneto, Tuscany, Apulia, and Sicily. The four chosen regions exemplify the four major Italian macro-areas, namely the North, Center, South, and Islands. Additionally, these regions have shown an increase in student outflows in recent times, with the leading universities in the northern regions, specifically situated in Lombardy, Piedmont, and Emilia-Romagna, emerging as the primary popular choice for students from these regions [4].

Table 1 provides a comprehensive description of the students attending high school in the aforementioned regions. This table includes their sociodemographic characteristics and high school performance. In addition, the number of clusters formed for each region is reported (see Sect 4.1 for details on the cluster construction). The data clearly reveals disparities between students from the northern regions, namely Veneto and Tuscany, and those hailing from the South and the Islands. On average, northern students exhibit higher maths scores and SES in comparison to their counterparts from the southern and island regions. Moreover, the percentage of students attending *licei* (including scientific and humanistic *licei*) is higher in Sicily and Apulia, whereas it falls below 50% in Veneto. Regarding the gender distribution, no significant differences are observed, as females make up around half of the student population in each region.

**Table 1:** Statistics of the sociodemographic characteristics and maths scores in Veneto, Tuscany, Apulia, and Sicily.

Region	Clusters	Maths score		SES		% Liceo	% Females
		mean	s.d.	mean	s.d.		
**Veneto**	15	219.41	36.99	0.07	0.96	44.9	51.6
**Tuscany**	18	207.92	39.71	0.13	0.99	54.3	50.8
**Apulia**	15	199.27	39.00	−0.10	1.04	57.4	52.7
**Sicily**	27	190.22	38.18	−0.01	1.02	60.4	52.3

**Fig 1:**
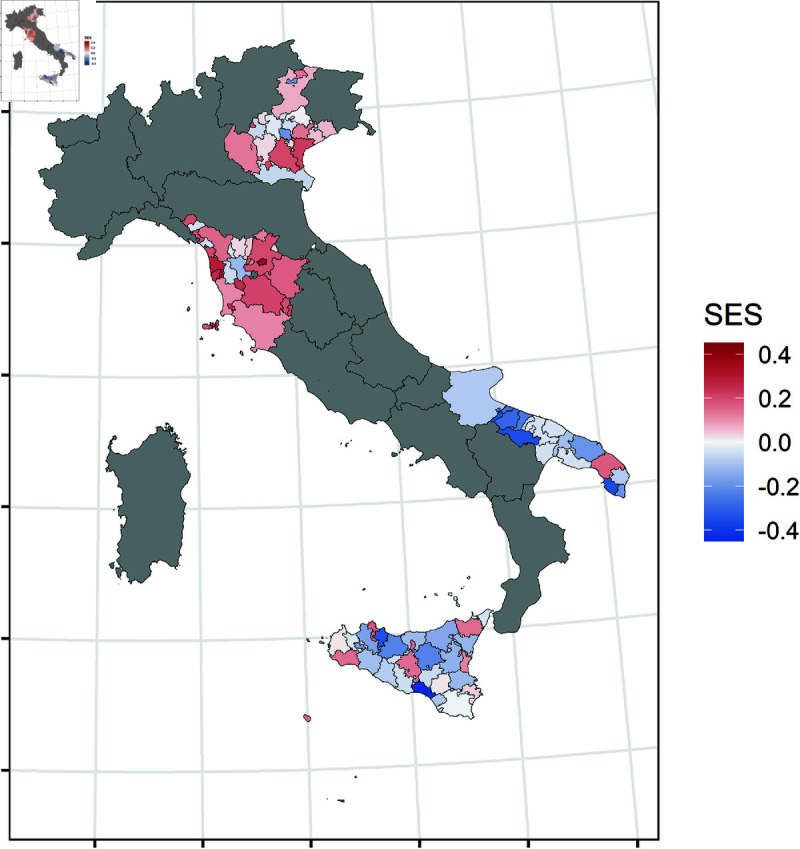
SES distribution in Veneto, Tuscany, Apulia, and Sicily at the cluster-level. The authors prepared this map using publicly available data from Istat https://www.istat.it/notizia/confini-delle-unita-amministrative-a-fini-statistici-al-1-gennaio-2018-2/, under the Creative Commons Attribution 4.0 International License (https://creativecommons.org/licenses/by/4.0/).

In Fig 1, the SES distribution at the cluster level is shown. The figure highlights the well-established divide between northern and southern Italian regions, providing a detailed overview of the SES heterogeneity across Italy. In detail, Tuscany and Veneto clusters exhibit a higher SES than the national average, while Apulia and Sicily display a lower SES. Notably, our analysis reveals that the clusters with a higher SES are mainly those hosting universities. This can be observed in Venice and Verona clusters for Veneto, Florence for Tuscany, and Lecce for Apulia. Finally, it is interesting to observe the Sicilian case, where the clusters with a university, i.e. Palermo, Catania, and Messina, show significantly higher SES levels than almost all the other clusters in the same region.

**Fig 2:**
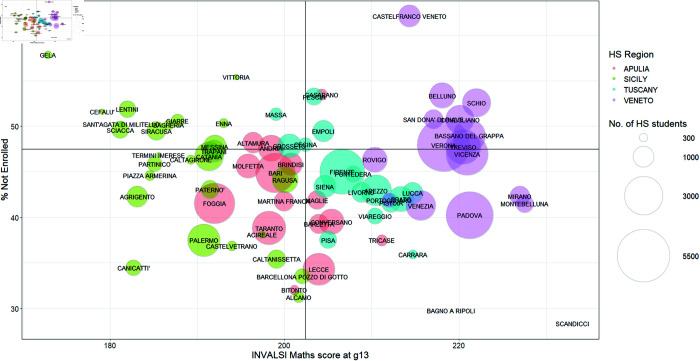
INVALSI maths score (X-axis) and % of not enrolled students (Y-axis) in Veneto, Tuscany, Apulia, and Sicily at the cluster-level. The size of the bubble corresponds to the number of students in each cluster.

**Fig 3:**
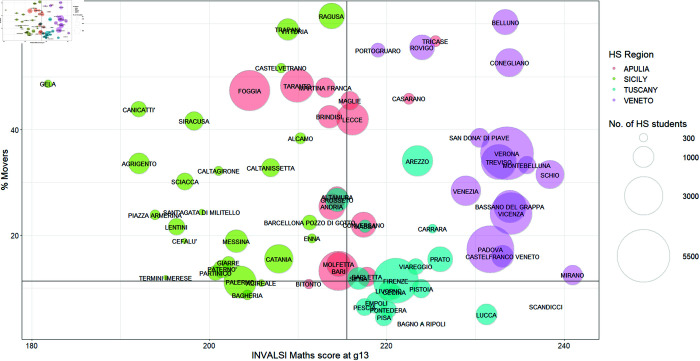
INVALSI maths score (X-axis) and % of mover students (Y-axis), in Veneto, Tuscany, Apulia, and Sicily at the cluster-level. The size of the bubble corresponds to the number of students in each cluster.

In Figs 2 and 3, the focus moves to the relationship between the scores in the INVALSI maths test and the two academic outcomes: i) university enrolment and ii) the choice to enrol outside the region where the student attended high school. Firstly, in Fig 2, we show the relationship between the INVALSI maths test scores and the percentage of not enrolled students by region at the cluster level. On average, individuals with higher maths scores show a higher probability of university enrolment in all regions.

In Fig 3, we see that the relationship between maths scores and the percentage of mover students has changed–we define a *mover* as a student who decides to enrol at a university in a region different from where he/she attended high school. This percentage is computed as the number of mover students over the total number of students enrolled at an Italian university in each cluster.

Overall, the results show a positive association between mathematics proficiency and academic outcomes. In northern regions, students with strong mathematical skills are more likely to attend universities within the region. This happens in Tuscany, where mobility rates are lower than in Veneto. However, it seems that, in Veneto, students with strong mathematical skills choose to enrol in universities outside their region, which is usually another northern region [4].

## 6 Results

In this section, we report the results of the balancing procedure (Table 2) and the estimated parameters of the multinomial models before and after the weighting procedure (Figs 4–11).

**Fig 4:**
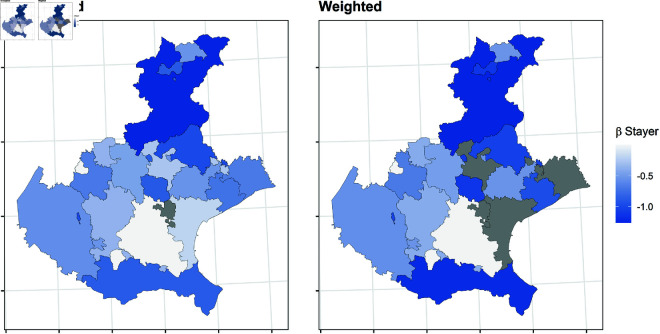
Estimated parameters of being *stayer* vs *not enrolled* before and after the weighting procedure for Veneto high school students. The authors prepared this map using publicly available data from Istat https://www.istat.it/notizia/confini-delle-unita-amministrative-a-fini-statistici-al-1-gennaio-2018-2/, under the Creative Commons Attribution 4.0 International License (https://creativecommons.org/licenses/by/4.0/).

**Fig 5:**
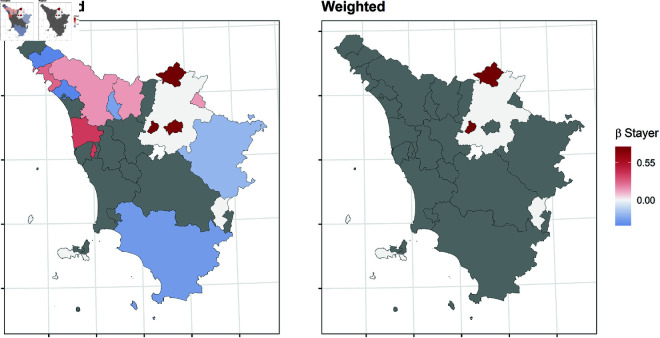
Estimated parameters of being *stayer* vs *not enrolled* before and after the weighting procedure for Tuscanian high school students. The authors prepared this map using publicly available data from Istat https://www.istat.it/notizia/confini-delle-unita-amministrative-a-fini-statistici-al-1-gennaio-2018-2/, under the Creative Commons Attribution 4.0 International License (https://creativecommons.org/licenses/by/4.0/).

**Fig 6:**
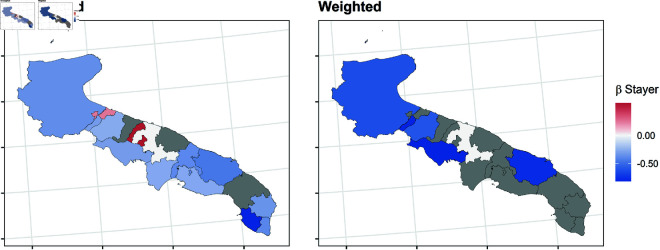
Estimated parameters of being *stayer* vs *not enrolled* before and after the weighting procedure for Apulian high school students. The authors prepared this map using publicly available data from Istat https://www.istat.it/notizia/confini-delle-unita-amministrative-a-fini-statistici-al-1-gennaio-2018-2/, under the Creative Commons Attribution 4.0 International License (https://creativecommons.org/licenses/by/4.0/).

**Fig 7:**
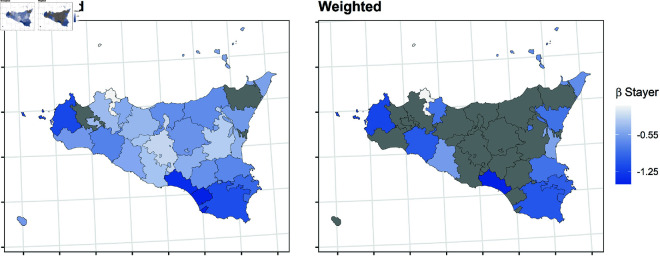
Estimated parameters of being *stayer* vs *not enrolled* before and after the weighting procedure for Sicilian high school students. The authors prepared this map using publicly available data from Istat https://www.istat.it/notizia/confini-delle-unita-amministrative-a-fini-statistici-al-1-gennaio-2018-2/, under the Creative Commons Attribution 4.0 International License (https://creativecommons.org/licenses/by/4.0/).

**Fig 8:**
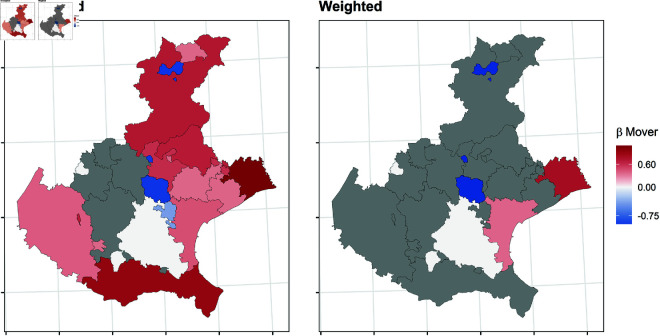
Estimated parameters of being *mover* vs *not enrolled* before and after the weighting procedure for Veneto high school students. The authors prepared this map using publicly available data from Istat https://www.istat.it/notizia/confini-delle-unita-amministrative-a-fini-statistici-al-1-gennaio-2018-2/, under the Creative Commons Attribution 4.0 International License (https://creativecommons.org/licenses/by/4.0/).

**Fig 9:**
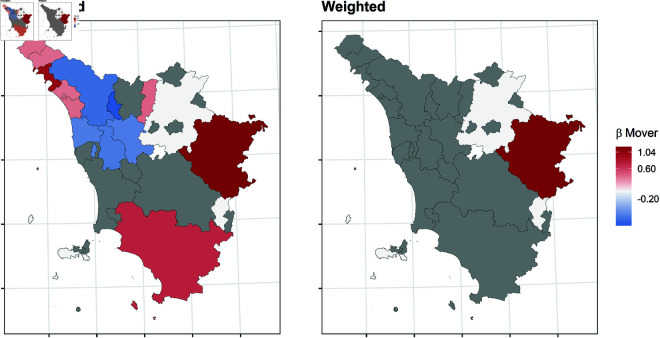
Estimated parameters of being *mover* vs *not enrolled* before and after the weighting procedure for Tuscanian high school students. The authors prepared this map using publicly available data from Istat https://www.istat.it/notizia/confini-delle-unita-amministrative-a-fini-statistici-al-1-gennaio-2018-2/, under the Creative Commons Attribution 4.0 International License (https://creativecommons.org/licenses/by/4.0/).

**Fig 10:**
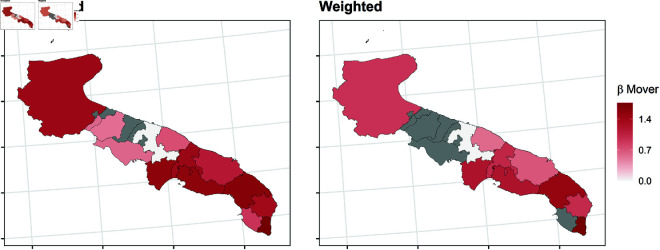
Estimated parameters of being *mover* vs *not enrolled* before and after the weighting procedure for Apulian high school students. The authors prepared this map using publicly available data from Istat https://www.istat.it/notizia/confini-delle-unita-amministrative-a-fini-statistici-al-1-gennaio-2018-2/, under the Creative Commons Attribution 4.0 International License (https://creativecommons.org/licenses/by/4.0/).

**Fig 11:**
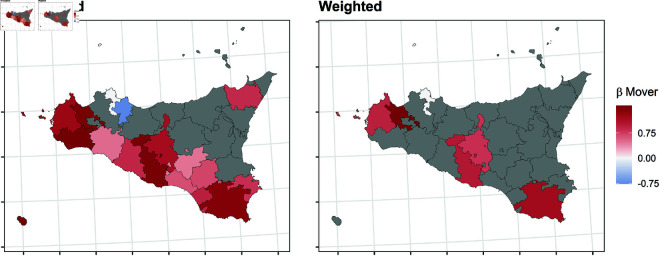
Estimated parameters of being *mover* vs *not enrolled* before and after the weighting procedure for Sicilian high school students. The authors prepared this map using publicly available data from Istat https://www.istat.it/notizia/confini-delle-unita-amministrative-a-fini-statistici-al-1-gennaio-2018-2/, under the Creative Commons Attribution 4.0 International License (https://creativecommons.org/licenses/by/4.0/).

**Table 2:** Maximum absolute standardized mean difference (ASMD), minimum p-value, and the number of significant differences observed in the one-to-one cluster comparisons before and after the weighting procedure.

Region	Variable	Unweighted				Weighted			
		Max ASMD	Min pvalue	Not Signif.	Signif.	Max ASMD	Min pvalue	Not Signif.	Signif.
**Veneto**	**Student SES**	0,44	0,00	29	76	0,05	0,27	105	0
	**Gender**	0,26	0,00	66	39	0,08	0,13	105	0
	**INVALSI maths score**	0,46	0,00	27	78	0,07	0,12	105	0
	**High school track:**								
	Hum/Sci licei	0,68	0,00	31	74	0,07	0,19	105	0
	Other licei	0,58	0,00	35	70	0,07	0,20	105	0
**Tuscany**	**Student SES**	0,47	0,00	66	87	0,15	0,26	153	0
	**Gender**	0,35	0,00	95	58	0,17	0,27	153	0
	**INVALSI maths score**	0,86	0,00	44	109	0,16	0,08	153	0
	**High school track:**								
	Hum/Sci licei	0,87	0,00	57	96	0,10	0,19	153	0
	Other licei	0,80	0,00	54	99	0,09	0,13	153	0
**Apulia**	**Student SES**	0,49	0,00	40	65	0,05	0,33	105	0
	**Gender**	0,26	0,00	57	48	0,14	0,06	105	0
	**INVALSI maths score**	0,49	0,00	41	64	0,07	0,19	105	0
	**High school track:**								
	Hum/Sci licei	0,64	0,00	29	76	0,13	0,12	105	0
	Other licei	0,57	0,00	36	69	0,08	0,17	105	0
**Sicily**	**Student SES**	0,59	0,00	165	186	0,17	0,18	351	0
	**Gender**	0,36	0,00	233	118	0,14	0,13	351	0
	**INVALSI maths score**	0,76	0,00	116	235	0,21	0,03	349	2
	**High school track:**								
	Hum/Sci licei	0,60	0,00	154	197	0,20	0,04	350	1
	Other licei	0,90	0,00	132	219	0,18	0,10	351	0

In Table 2, the overall summary measures of balance are obtained by taking the maximum absolute standardized mean difference (ASMD), and the minimum p-values observed in the one-to-one cluster comparisons. Additionally, we report the number of significant and non-significant differences observed in the one-to-one cluster comparisons within each region, before and after the weighting procedure. Results show the procedure perfectly balances the clusters for Apulia, Tuscany, and Veneto. In Sicily, there are some imbalances corresponding to the cluster of Gela, where the balancing procedure fails because of the lower percentage of students attending humanities and scientific *licei* compared to the other Sicilian clusters.

Figs 4–11 show the *β*’s estimated for the two equations of the multinomial logistic regression models (Eqs 2 and 3) before (left panel) and after (right panel) the weighting procedure (see Tables 3 and 4 for the values of the estimated *β*’s). The reference cluster for each region is set where the largest university is located. For the sake of clarity, we adopted different colour scales for the *β*’s in each region.

The interpretation of the *β*’s is two-fold for both outcomes. The first one (*i*) pertains to the differences between the unweighted and the weighted *β*’s: big (small) differences correspond to clusters where the adopted procedure was (was not) necessary; in other words, the covariates included in the balancing procedure hid (did not hide) the territorial effect. The second one (*ii*) pertains to the interpretation typically ascribed to the *β*’s, which measure the difference with the reference cluster on the outcomes. In other words, the significance of the *β* after the weighting procedure indicates that the difference with the reference cluster can be reasonably attributable to the territory.

Figs 4–7 present the estimated $\beta_{1}$’s for being a *stayer* compared to *not enrolled*.

*(i)* The weighting procedure resulted in noticeable changes for both outcomes, indicating the territorial effect may have been concealed or counterbalanced by other student characteristics. Specifically, the effect of the other covariates was stronger in some clusters, leading to the non-significance of the estimated $\beta_{1}$’s associated with those clusters. This is more evident in Tuscany, where most $\beta_{1}$’s are no longer significant. On the contrary, the territorial effect appears to be more pronounced in Veneto. As for Apulia and Sicily, the procedure reveals that the territorial effect is present only in some clusters, indicating that the effect of the other covariates was stronger.
*(ii)* Moreover, the procedure revealed an even stronger effect for those clusters in which a significant $\beta_{1}$ persists. In Veneto, the $\beta_{1}$’s suggest all the clusters produce a deterring effect to enrol in the same region, especially in the northern and southern clusters, which are farther from the university cities. Conversely, the weighted $\beta_{1}$’s indicate no territorial effect on the choice to enroll in Tuscany, except for Scandicci, a cluster adjacent to Florence. Concerning southern regions, in Apulia, the $\beta_{1}$’s for the southern clusters of the region are no more significant (except for Brindisi), while they have decreased in northern clusters; in Sicily, the $\beta_{1}$’s for the inner areas are no more significant, while they have decreased especially in the southern-coast clusters, like Sciacca.

Focusing now on the estimated $\beta_{2}$’s of being mover vs not enrolled, Figs 8–11 show some differences from the results observed in the previous transition.

*(i)* The weighting procedure has produced big differences in the $\beta_{2}$’s. This is evident in Veneto and Tuscany, where most $\beta_{2}$’s are no longer significant, highlighting the role of students’ characteristics in the choice to move to another region. As for Apulia, the probability of moving to another region is still significantly higher even in the clusters hosting a university, such as Lecce and Foggia. This is probably because these two universities were recently founded and do not offer many courses. In Sicily, the territorial effect is still present in the southern-central area and the Trapani and Ragusa areas. Trapani is, in some way, a unique case. Despite being quite close to the largest Sicilian university, Palermo, it maintains an exceptionally high probability of moving outside of Sicily.
*(ii)* It is worth specifying that the significance of the $\beta_{2}$’s in Veneto and Tuscany is attributable to mobility towards adjacent regions: 74.6% of the movers from the Portogruaro cluster (Veneto) enroll in Friuli-Venezia-Giulia, while 68.4% from the Arezzo cluster (Tuscany) enroll in Umbria and Emilia-Romagna. On the other hand, mobility flows from Apulia and Sicily are almost entirely directed to central and northern regions. In Apulia, where the territorial effect is still pronounced after the weighting procedure, the balancing covariates do not play an important role. In Sicily, the pattern observed in the first transition persists, as Trapani, central-southern Sicily, and Ragusa are confirmed to be areas experiencing significant mobility flows.

## 7 Conclusions

Inequalities in educational opportunities are related to several factors, such as socioeconomic status, previous educational outcomes, and territory. The Italian case is particularly interesting because of the distinctive pattern of disparities among the country’s areas and the persistence over the decades of migration flows from the southern regions to the richest ones in the North. In this regard, this work aimed to study the territorial effect on the transition from high school to university in four Italian regions, i.e. Veneto, Tuscany, Apulia, and Sicily, each one representing the four macro-areas of the country (North, Center, South, and Islands). To isolate the territorial effect within each region, we tried to balance clusters of municipalities for gender, socioeconomic status, and previous educational attainment, intended as the scores in INVALSI maths tests and the type of high school track attended. To do this, we used a propensity score procedure based on GBM, which provided an almost perfect balance of the clusters within each region. Then, we used the inverse of the propensity scores as weights of four multinomial logistic regression models, one for each region, to estimate the cluster differences in enrolment choices, and compared the results before and after the weighting procedure.

There are two possible interpretations of the results from a territorial perspective: the first concerns absolute values related to mobility rates among the different regions, while the second concerns the relationship between the reference cluster (Padua, Florence, Bari, Palermo) and the other clusters within their respective regions. The results of the multinomial models highlight indeed different mobility patterns within each region. After the balancing procedure, the results show that the $\beta_{s}$ associated with the choice to stay in the same region (versus not enrolling) are always negative compared to the reference cluster. In particular, this occurs in Veneto and Apulia, where most differences are still significant after the weighting procedure, highlighting how territory effects do not interact with the other covariates.

Regarding the choice to enrol in another region, as expected, southern regions have higher mobility rates. In particular, the territorial effect appears to be stronger in Apulia, which could be explained by the recent establishment of the universities in that region, except for the one located in Bari. In contrast, mobility patterns in northern regions primarily consist of mobility to adjacent areas, which is not “real” mobility since it involves commuting students.

Moreover, the results highlight a more pronounced territorial heterogeneity regarding the choice to move to another region compared to northern regions. While Sicily and Apulia share high mobility rates, the significant territorial effect in Apulia can be attributed to two factors: firstly, the observed differences with the reference cluster Bari, with a mobility rate of approximately 7% (considering non-enrolled students in the calculation), may be due to the smaller size of universities recently established in those areas. Secondly, Apulia benefits from better connectivity with other Italian regions, a crucial territorial aspect to consider when contemplating the idea of moving to another region. Additionally, the data show the higher heterogeneity of the Sicilian territory in terms of mobility rates, ranging from 8.6% in Bagheria (near Palermo) to 61.5% in Ragusa.

Overall, the study highlights the importance of considering the complex interplay between territory and economic variables in explaining inequalities in the transition from high school to university. Particularly, our procedure helped us to isolate the territorial effect on mobility choices, showing how this effect, especially in northern regions, was covered by socioeconomic status and previous educational attainment. The causes of regional differences in enrolment choices have indeed to be sought outside students’ sociodemographic and educational backgrounds, taking into account the role of those contextual factors that affect their educational choices and attainment. It is reasonable to imagine that differences in enrolment choices are influenced by a region’s economic and social context. Regions with a strong economic structure may provide better educational and career opportunities to their students, potentially leading to higher university enrolment rates. Furthermore, regional heterogeneity in enrollment choices may also be related to territorial disparities in access to educational resources. For instance, students from inner southern areas experience significant difficulties related to poor infrastructure and difficulty in reaching educational institutions. On the other hand, the presence of well-established educational infrastructures, such as in most central and northern regions, may contribute to creating an environment that boosts students’ aspirations and academic pursuits.

The territorial and contextual influence potentially encompasses cultural and social dimensions that can have a significant impact on students’ educational paths. On the one hand, the decision not to attend university can, therefore, also reflect a deep-rooted territorial identity, where the value placed on local traditions and responsibilities outweighs the potential benefits of higher education. Conversely, for those who choose to leave — particularly individuals from southern regions as the results indicate — the phenomenon of “brain drain” is often deeply tied to a fragmented sense of belonging. While local identity retains its importance, it ultimately proves to be insufficient to outweigh practical considerations. In this respect, when certain professions are highly respected or traditional vocational paths are prevalent in a specific area, students may prefer to follow these established trajectories, contributing to the observed regional variations in university enrollment rates.

As future development, it would be interesting to repeat the analysis considering the whole country and taking into account also macro-level variables regarding the macro-level territorial characteristics.

Moreover, future research could focus on a detailed analysis of how the pandemic has influenced the educational choices of Italian students. According to findings by [55], there was an increase in university enrollments, but there was also a substantial reduction in outgoing mobility from Southern Italy. It would be interesting to assess whether the pandemic affected students’ geographical origins in this context. The shift to remote learning at universities may have encouraged enrollment among students who might not have considered higher education otherwise. On one hand, online education helped reduce the socioeconomic gap between different regions by lowering the costs associated with both national and intra-regional mobility. On the other hand, the suspension of social and economic activities may have led students to enroll in university since there were limited alternatives for entering the labor market quickly.

## Appendix

**Table 3:** Parameters estimated from the multinomial model for the two equations (Veneto and Tuscany).

Region	Cluster	Stayer vs not enrolled		Mover vs not enrolled	
		Unweighted	Weighted	Unweighted	Weighted
**TUSCANY**	**FIRENZE (ref.)**				
	**AREZZO**	−0,211	−0,104*	1,181	1,180
	**BAGNO A RIPOLI**	0,746	1,009*	−0,609*	−0,276*
	**CARRARA**	0,261	0,253*	1,003	0,915*
	**CECINA**	−0,096*	−0,246*	−0,332*	−0,417*
	**EMPOLI**	−0,132*	−0,283*	−0,639	−0,934*
	**GROSSETO**	−0,304	−0,392*	0,739	0,546*
	**LIVORNO**	0,113*	0,180*	−0,092*	−0,025*
	**LUCCA**	0,165	0,317*	−0,738	−0,576*
	**MASSA**	−0,376	−0,525*	0,394	0,097*
	**PESCIA**	−0,274	−0,505*	−0,918	−1,038*
	**PISA**	0,385	0,126*	−0,635	−0,666*
	**PISTOIA**	0,157	0,158*	−0,006*	−0,025*
	**PONTEDERA**	0,072*	−0,049*	−0,658	−0,915*
	**PRATO**	0,075*	0,231*	0,429	0,567*
	**SCANDICCI**	0,782	1,121	0,162*	1,36*
	**SIENA**	0,062*	0,048*	0,102*	0,038*
	**VIAREGGIO**	0,168*	0,369*	0,410	0,528*
**VENETO**	**PADOVA (ref.)**				
	**BASSANO DEL GRAPPA**	−0,457	−0,543	0,030*	−0,150*
	**BELLUNO**	−1,263	−1,460	0,719	0,527*
	**CASTELFRANCO VENETO**	−0,873	−1,270	−0,969	−1,571
	**CONEGLIANO**	−0,981	−1,199	0,689	0,368*
	**MIRANO**	−0,029*	0,053*	−0,417	−0,478*
	**MONTEBELLUNA**	−0,266	−0,207*	0,601	0,553*
	**PORTOGRUARO**	−0,675	−0,509*	1,088	1,169
	**ROVIGO**	−0,868	−1,394	0,918	0,444*
	**SAN DONA’ DI PIAVE**	−0,721	−1,106	0,373	−0,161*
	**SCHIO**	−0,685	−0,779	0,099*	−0,056*
	**TREVISO**	−0,527	−0,601	0,367	0,275*
	**VENEZIA**	−0,186	−0,224*	0,450	0,541
	**VERONA**	−0,560	−0,646	0,406	0,278*
	**VICENZA**	−0,358	−0,457	0,058*	−0,128*

**Table 4:** Parameters estimated from the multinomial model for the two equations (Apulia and Sicily).

Region	Cluster	Stayer vs not enrolled		Mover vs not enrolled	
		Unweighted	Weighted	Unweighted	Weighted
**APULIA**	**BARI (ref.)**				
	**ALTAMURA**	−0,309	−0,693	0,592	0,086*
	**ANDRIA**	−0,261	−0,502	0,547	0,185*
	**BARLETTA**	0,242	0,245*	0,143*	0,064*
	**BITONTO**	0,574	0,083*	0,345*	−0,706*
	**BRINDISI**	−0,449	−0,654	1,131	0,853
	**CASARANO**	−0,817	−0,971*	0,902	0,652*
	**CONVERSANO**	0,114*	0,118*	0,729	0,708
	**FOGGIA**	−0,365	−0,524	1,415	1,146
	**LECCE**	0,042*	0,097*	1,602	1,812
	**MAGLIE**	−0,344	−0,439*	1,359	1,206
	**MARTINA FRANCA**	−0,386	−0,507*	1,417	1,243
	**MOLFETTA**	−0,046*	−0,272*	0,063*	0,008*
	**TARANTO**	−0,269	−0,279*	1,548	1,496
	**TRICASE**	−0,392	−0,257*	1,765	2,193
**SICILY**	**PALERMO (ref.)**				
	**ACIREALE**	−0,023*	−0,312*	−0,046*	−0,570*
	**AGRIGENTO**	−0,490	−0,640	0,905	0,436*
	**ALCAMO**	−0,086*	0,138*	1,516	1,575
	**BAGHERIA**	−0,489	−0,951	−0,771	−0,918*
	**BARCELLONA POZZO DI GOTTO**	0,039*	0,129*	0,873	0,951*
	**CALTAGIRONE**	−0,634	−0,563*	0,698	0,932*
	**CALTANISSETTA**	−0,189	−0,290*	1,170	0,829
	**CANICATTI’**	−0,326	−0,617*	1,503	1,022
	**CASTELVETRANO**	−0,582	−1,139*	1,564	1,278*
	**CATANIA**	−0,428	−0,587	−0,048*	−0,237*
	**CEFALU’**	−0,666	−1,493*	−0,046*	−1,098*
	**ENNA**	−0,621	−0,566*	0,032*	−0,105*
	**GELA**	−1,376	−1,696	0,649	−0,054*
	**GIARRE**	−0,577	−0,968	−0,253*	−0,909*
	**LENTINI**	−0,711	−0,951	0,076*	−0,411*
	**MESSINA**	−0,512	−0,769	0,105*	−0,361*
	**PARTINICO**	−0,363	−0,631*	−0,205*	−0,403*
	**PATERNO’**	−0,259	−0,523*	−0,031*	−0,613*
	**PIAZZA ARMERINA**	−0,447	−0,713*	0,468	0,080*
	**RAGUSA**	−1,110	−1,189	1,436	1,203
	**SANT’AGATA DI MILITELLO**	−0,681	−1,118*	0,264*	−0,781*
	**SCIACCA**	−0,735	−1,166	0,502	−0,240*
	**SIRACUSA**	−0,910	−1,115	0,830	0,403*
	**TERMINI IMERESE**	−0,393	−0,995*	−0,314*	−1,485*
	**TRAPANI**	−1,172	−1,339	1,267	0,954
	**VITTORIA**	−1,492	−1,929*	0,934	0,257*
